# Effect of Patient Age on Timing of Inpatient Esophagogastroduodenoscopy and Outcomes for Non-variceal Upper GI Bleeds

**DOI:** 10.7759/cureus.39302

**Published:** 2023-05-21

**Authors:** Anmol Mittal, Faiz Afridi, Ayham Khrais, Sushil Ahlawat

**Affiliations:** 1 Internal Medicine, Rutgers University New Jersey Medical School, Newark, USA; 2 Gastroenterology and Hepatology, Rutgers University New Jersey Medical School, Newark, USA

**Keywords:** upper gi bleed, acute gi bleed, egd timing, geriatrics, esophagogastroduodenoscopy (egd)

## Abstract

Background

Esophagogastroduodenoscopy (EGD) is typically performed within 24 hours of presentation for patients admitted to a hospital for patients presenting with a non-variceal upper gastrointestinal bleed (UGIB). To date, no studies have been performed to identify the impact of patient age on the timing of inpatient EGD and patient outcomes in non-variceal UGIB. Our aim was to assess the differences in the timing of EGD, blood transfusion requirements, development of hemorrhagic shock, development of acute renal failure, mortality, length of stay, and total hospital charges for patients aged 18-59 and those aged 60 and older.

Methods

Admissions for non-variceal UGIB were identified from the National (Nationwide) Inpatient Sample (NIS) database from 2016 and 2017. Patients who initially presented with hemorrhagic shock were excluded. Patients were divided into two age groups, those aged 18-59 and those aged 60 or older. We classified EGDs as early and delayed. Since the NIS database identifies days as midnight to midnight, we categorized early EGDs as those performed on day 0 and day 1. Delayed EGD were categorized as those performed on days 2 and 3. Multivariate logistic regression was performed on propensity-matched data to compare EGD timing, blood transfusion requirements, development of post-hospitalization hemorrhagic shock, development of acute renal failure, and mortality. The following patient and hospital variables were used in regression models: race, sex, insurance status, income quartile, mortality risk score, illness severity score, admission month, admission day, type of admission, region, bed size, and hospital teaching status. Finally, weighted two-sample T-tests were used to compare the length of stay and total hospitalization cost.

Results

A total of 12,449 weighted cases of inpatient non-variceal UGIB were included in this study. Patients aged 60 and older were more likely to die during the hospitalization (OR= 1.661, 95%CI: 1.108-2.490, p= 0.014), require blood transfusion (OR= 1.257, 95%CI: 1.131-1.396, p<0.001), and develop acute renal failure (OR= 1.672, 95%CI: 1.447-1.945, p<0.001). Patients aged 60 and older were also less likely to receive an early EGD (OR= 0.850, 95%CI: 0.752-0.961, p= 0.009). Total hospital costs (95%CI: -1397.77 - -4005.68, p<0.001) and length of stay (95%CI: -0.428 - -0.594, p<0.001) were both lower in patients aged 18-59 years. There was no difference in the development of post-hospitalization hemorrhagic shock between the two groups (OR= 0.984, 95%CI: 0.707-1.369, p= 0.923).

Conclusions

Patients aged 60 and older were less likely to have an early EGD and more likely to have worse outcomes. They had increased rates of inpatient mortality, blood transfusion requirements, development of acute renal failure, increased total hospital costs, and longer lengths of stay. There were no differences in the development of post-hospitalization hemorrhagic shock between the two groups.

## Introduction

Upper gastrointestinal bleed (UGIB) is defined as bleeding proximal to the ligament of Treitz [1]. It can present as hematemesis, melena, or less commonly as hematochezia [2]. UGIB is most commonly due to peptic ulcer disease, esophageal or gastric varices, and erosive esophagitis [2]. It represents a significant strain on the healthcare system, accounting for 400,000 admissions annually and one billion dollars in annual spending [1,3]. On average, each hospitalization for UGIB costs $11,228 while 12-month spending on such patients totals $20,405 [4].

Geriatric patients are especially susceptible to significant morbidity and mortality from UGIB. Geriatric UGIB is largely secondary to peptic ulcer disease, driven by the use of aspirin and anticoagulants for the management of other comorbidities [1]. Mortality from UGIB ranges from 4% to 14%, driven primarily by older patients with multiple comorbidities. Mortality in patients under the age of 60 is approximately 0.4% but increases to 11% in patients over the age of 80 [3].

Numerous studies have demonstrated disparities in care for older patients [5-7]. However, these disparities have typically been associated with a greater number of underlying comorbidities in elderly patients rather than the decision-making level [5-8]. Therefore, we created this study to compare and evaluate differences in time to esophagogastroduodenoscopy (EGD) for non-variceal UGIB in patients 18-59 years old and 60 years or older. We also chose to evaluate the following patient-related outcomes: in-hospital mortality, blood transfusion requirement, development of hemorrhagic shock, and development of acute renal failure. Finally, we compared the length of stay and total charges between the two groups.

We expected patients in the older age group to have worse outcomes, consistent with previous studies [5-7]. However, due to the American College of Gastroenterology (ACG) recommendation of an EGD to evaluate UGIB within 24 hours for all hospitalization patients, we expected no difference in time to EGD when hospital and patient factors were controlled [9]. Thus, we expected no association between decision-making in the care of elderly patients admitted for UGIB and in-hospital outcomes.

This article was previously presented as a meeting abstract at the 2022 Digestive Diseases Week Annual Scientific Meeting on May 22, 2022 [10].

## Materials and methods

We performed a retrospective review of inpatient admissions from 2016 and 2017 using the National (Nationwide) Inpatient Sample (NIS) database. The database is representative of 20% of hospitalizations in the United States. It contains up to 30 discharge diagnoses and 15 procedural codes for each admission. The database records patient demographics, insurance status, total cost of hospitalization, length of stay, and patient outcomes. It is maintained by the Healthcare Cost and Utilization Project (HCUP) of the Agency for Healthcare Research and Quality. The NIS database has been shown to correlate with other large databases, such as the National Hospital Discharge Survey, and has been used in previous studies [11-14]. The NIS database provides a "discharge weight" to approximate a national estimate, a correction that accounts for the methodology by which NIS obtains the data. The unweighted data accounts for about seven million hospital stays, after applying the weighted variable provided by NIS, over 35 million hospital stays are represented. 

Study population

We first identified patients 18 years or older who were admitted for a non-variceal upper GI bleed using International Classification of Diseases, 10th Revision (ICD-10) codes (K20.81, K20.91, K21.01, K22.1, K25.0, K25.4, K26.0, K26.4, K27.0, K27.4, K29.01, K29.31, K29.41, K29.51, K29.61, K29.71, K29.81, K29.91, K31.811, K31.82). NIS classifies the first two diagnosis codes as the reason for hospital admission [15,16]. We eliminated patients who were admitted for variceal bleed using ICD-10 codes (I85.01, I85.11) and hemorrhagic shock (R57.1) due to the urgent need for endoscopic intervention. EGD was identified by using the ICD-10 procedure codes. The codes for inpatient EGD were 0DJ07ZZ and 0DJ08ZZ.

Study variables/outcomes

The purpose of this study was to compare the timing and outcomes of inpatient EGDs in patients aged 18-59 and those aged 60 and older. Our primary outcome was to evaluate if there was a difference in the timing of EGDs in the two age groups. Secondary outcomes included mortality, blood transfusion requirements, development of post-hospitalization hypovolemic shock, development of acute renal failure, length of stay, and total cost of hospitalization. The Healthcare Cost and Utilization Project of the Agency for Healthcare Research and Quality defines a teaching hospital as a hospital with an Accreditation Council for Graduate Medical Education (ACGME)-accredited residency program and/or the presence of interns with a resident-to-patient ratio of >0.25 [11].

Statistical analysis

Statistical analysis was performed using IBM SPSS Statistics for Windows, Version 28.0 (IBM Corp., Armonk, New York, United States). Multivariate logistic regressions using propensity-matched data were built to evaluate the timing of EGD, mortality, blood transfusion requirements, development of post-hospitalization hypovolemic shock, and development of acute renal failure in patients aged 18-59 and those aged 60 and older. The following patient and hospital variables were used in regression models: race, sex, insurance status, income quartile, mortality risk score, illness severity score, admission month, admission day, type of admission, region, hospital bed size, and hospital teaching status. A weighted two-sample T-test was used to compare the length of stay and total cost of hospitalization between the two groups. A p-value less than 0.05 was considered statistically significant.

Regression analysis was based on encounters without missing values. An odds ratio (OR) whose entire 95% confidence interval (CI) is greater than one indicated a greater likelihood of that event when compared to the reference event. Conversely, an OR whose entire 95%CI is less than one indicates lower odds of the event when compared to the reference event. An OR whose 95%CI contains one indicates no difference when compared to the reference event.

## Results

A total of 12,449 cases of inpatient non-variceal UGIB were included in this study. Of this, 3,753 patients were aged 18-59 while 8,696 patients were 60 or older. Of the 18-59-year-old patients, 2,988 underwent an EGD, while 6,477 of the 60 years or older patients underwent EGD. These numbers represented a 20% sample of all non-variceal UGIB in the United States over the study period. Patients aged 60 and older were less likely to undergo early EGD (OR= 0.850, 95%CI: 0.752-0.961, p= 0.009) (Table 1). This age group was more likely to die during the hospitalization (OR= 1.661, 95%CI: 1.109-2.490, p= 0.014), require a blood transfusion (OR= 1.257, 95%CI: 1.131-1.396, p<0.001), and develop acute renal failure (OR= 1.672, 95%CI: 1.447-1.945, p<0.001) (Table 1). Total hospital costs (95%CI: -1397.77 - -4005.68, p<0.001) and length of stay (95%CI: -0.428 - -0.594, p<0.001) were lower in patients aged 18-59 ) (Table 2). No difference was seen in the development of hypovolemic shock during the hospital stay between the two groups (OR= 0.985, 95%CI: 0.707-1.369, p=0.923) (Table 1).

**Table 1:** Differences in timing of esophagogastroduodenoscopy and patient outcomes among patients aged 18-59 and those aged 60 and older via logistic regression analysis of propensity-matched data

	Odds ratio (95% CI)	p-value
Mortality		
18-59	REF	REF
60+	1.661 (1.108-2.490)	0.014
Early EGD		
18-59	REF	REF
60+	0.850 (0.752-0.961)	0.009
Blood transfusion		
18-59	REF	REF
60+	1.257 (1.131-1.396)	<0.001
Acute renal failure		
18-59	REF	REF
60+	1.672 (1.447-1.945)	<0.001
Hypovolemic shock		
18-59	REF	REF
60+	0.984 (0.707-1.369)	0.923

**Table 2:** Differences in total charges and length of stay among patients aged 18-59 and those aged 60 and older via weighted two-sample T-test

	95% Confidence interval	p-value
Total charges	-1397.77 - -4005.68	<0.001
Length of stay	-0.428 - -0.594	<0.001

Patient demographics

Patients aged 60 and older that were included in the study were most likely to be Caucasian or Asian/Pacific Islander (OR = 1.120, 95%CI: 0.840-1.490, p = 0.440) than African American (OR = 0.594, 95%CI: 0.507-0.695, p <0.001), Hispanic (OR = 0.690, 95%CI: 0.575-0.827, p <0.001) or Native American (OR = 0.368, 95%CI: 0.196-0.691, p = 0.002) (Table 3). This age demographic was also most likely to have an income of $45,000 or more (OR = 1.326, 95%CI: 1.133-1.552, p <0.001) (Table 3). They were also most likely to be insured by Medicare when compared to other insurance carriers. Patients aged 60 or older were also more likely to be female (OR = 1.256, 95%CI: 1.199-1.315, p <0.001) (Table 3). Finally, none of the mortality risk scores and illness severity scores were more likely to be associated with patients aged 60 years or older (Table 3). 

**Table 3:** Hospital and patient characteristics most likely to be associated with patients aged 60 and older

Variable	P-value	Odds ratio (95% confidence interval)
Admission month		
January	REF	REF
February	0.690	1.052 (0.820-1.350)
March	0.054	1.272 (0.996-1.625)
April	0.037	1.305 (1.017-1.676)
May	0.474	1.094 (0.855-1.401)
June	0.153	1.201 (0.934-1.545)
July	0.316	1.137 (0.855-1.461)
August	0.104	1.230 (0.958-1.580)
September	0.099	1.233 (0.961-1.581)
October	0.356	1.123 (0.878-1.436)
November	0.436	1.103 (0.862-1.410)
December	0.060	1.266 (0.990-1.619)
Admission day		
Weekday	REF	REF
Weekend	0.107	0.909 (0.808-1.021)
Type of admission		
Elective	REF	REF
Non-elective	0.034	1.350 (1.024-1.781)
Region		
New England	REF	REF
Mid-Atlantic	0.211	1.187 (0.907-1.553)
East North Central	0.967	0.995 (0.773-1.279)
West North Central	0.797	1.040 (0.773-1.400)
South Atlantic	0.983	0.997 (0.779-1.277)
East South Atlantic	0.013	0.696 (0.522-0.928)
West South Atlantic	0.530	1.091 (0.832-1.431)
Mountain	0.819	1.035 (0.772-1.386)
Pacific	0.287	1.147 (0.891-1.477)
Bedsize		
Small	REF	REF
Medium	0.404	1.066 (0.918-1.238)
Large	0.665	0.970 (0.845-1.114)
Teaching status		
Non-teaching	REF	REF
Teaching	0.781	1.016 (0.910-1.133)
Mortality risk score		
Minor likelihood of dying	REF	REF
Moderate likelihood of dying	0.859	0.776 (0.047-12.712)
Major likelihood of dying	0.874	0.797 (0.049-13.005)
Extreme likelihood of dying	0.974	1.047 (0.064-16.990)
Race		
Caucasian	REF	REF
African American	<0.001	0.594 (0.507-0.695)
Hispanic	<0.001	0.690 (0.575-0.827)
Asian or Pacific Islander	0.440	1.120 (0.840-1.490)
Native American	0.002	0.368 (0.196-0.691)
Illness severity score		
Minor loss of function	REF	REF
Moderate loss of function	0.458	0.887 (0.646-1.218)
Major loss of function	0.500	0.902 (0.667-1.218)
Extreme loss of function	0.984	1.003 (0.763-1.218)
Median income		
$1-24,999	REF	REF
$25,000-34,999	0.729	1.025 (0.893-1.177)
$35,000-44,999	0.236	1.091 (0.944-1.261)
$45,000 or more	<0.001	1.326 (1.133-1.552)
Insurance status		
Medicare	REF	REF
Medicaid	<0.001	0.032 (0.028-0.037)
Private insurance	<0.001	0.076 (0.068-0.086)
Self-pay	<0.001	0.028 (0.022-0.036)
No charge	<0.001	0.022 (0.010-0.050)
Other insurance status	<0.001	0.093 (0.072-0.120)
Sex		
Male	REF	REF
Female	<0.001	1.256 (1.199-1.315)

## Discussion

In this study, we demonstrated that patient age impacts time to EGD in non-variceal UGIB, along with in-hospital mortality, blood transfusion requirements, development of acute renal failure, length of stay, and total charges. Current guidelines recommend an EGD within 24 hours to evaluate a non-variceal UGIB [9]. Further, American Society for Gastrointestinal Endoscopy (ASGE) recommendations from 2013 do not alter this guideline for older patients if the patient is medically fit for endoscopic interventions [17]. Therefore, while we expected worse in-hospital outcomes for older patients, we hypothesized that there would be no differences in time to EGD for patients aged 60 years or older.

The efficacy and safety of EGD in elderly patients have been demonstrated in the past. In one study of patients aged 65-89, diagnostic information from EGD was obtained in 93% when evaluating all conditions while another study evaluating EGD for UGIB demonstrated a 74% yield [17,18]. While studies have demonstrated that older patients are more likely to have lower blood pressures, decreased oxygen saturation, and increased atrial pressures when compared to their younger counterparts during EGD, these findings did not result in any clinically meaningful events [18]. As a result of these findings, the ASGE recommends standard intra-operative procedures including monitoring devices, resuscitation equipment, and pharmacologic agents for all age groups [17]. They do note that physicians should have increased awareness of sedative effects on the elderly and spend more time in preoperative evaluation in older patients to identify suitable candidates for endoscopy [17].

We, therefore, chose to study outcomes in those who presented with a non-variceal UGIB and underwent an EGD by admission day 3. We used day 3 as a surrogate for 72 hours since admission. This limited bias from patients who were deemed not to be fit for EGD or those whose UGIB was not significant enough to necessitate an earlier EGD. As expected, we found worse inpatient outcomes for patients above the age of 60. They were more likely to die in the hospital, require blood transfusions, and develop acute renal failure than younger patients. Furthermore, their healthcare utilization resource usage was greater than those patients aged 18-59 with longer lengths of stay and higher total charges. Unexpectedly, however, patients above the age of 60 were less likely to receive an early EGD than patients aged 18-69.

In recent years, a growing number of calls have been made for healthcare reform to address disparities in care. These disparities have typically revolved around vulnerable underinsured patients and those belonging to ethnic minorities. Abougergi et. al. demonstrated that Black and underinsured patients had worse outcomes and greater resource utilization in non-variceal UGIB [19]. Disparity in care for vulnerable populations has been shown to be widespread in medicine and prevalent amongst diverse specialties and subspecialties [14,20-22]. These studies have included elderly patients as a group with worse outcomes but have typically associated these outcomes with underlying comorbidities. Our study demonstrates that the effect of patient’s age on time to EGD and worse outcomes was independent of patient comorbidities. These findings highlight the disparity in both decision-making and outcomes for elderly patients. We argue that healthcare reform can only be successful if biases of care for elderly patients are addressed.

Our call will be especially important as demographic shifts engulf the United States over the coming decades. On a societal level, implicit and explicit healthcare biases against the elderly will affect larger proportions of the population. By 2030, there will be more Americans above the age of 65 than under the age of five and more than 20% of the American population will be above the age of 65 [1,23]. Furthermore, healthcare spending will also continue to increase with this aging population. Our data demonstrated that patients aged 18-59 had lower in-hospital costs, with 95%CI between 1397.77 and 4005.68 dollars less, and lower lengths of stay, with 95%CI between 0.428 to 0.594 days shorter. These findings are especially concerning given that UGIB primarily occurs in patients over the age of 65 and the rapid growth in United States healthcare spending as a share of the growth domestic product [1,23].

Our study has several limitations common with database studies. We relied on the assumption that each entry was a unique patient. We could not account for patients who were readmitted because of non-variceal UGIB or duplicate entries into the system. Furthermore, as a retrospective study, we could not account for all confounders. However, we did try to limit them by performing multivariate logistic regressions controlling for patient and hospital-related characteristics. Furthermore, we were reliant on the integrity of the NIS database for our results. This relies on the assumption that accurate coding is done at the time of discharge. Finally, it should be noted that given the nature of this retrospective study, we can only draw associations between delayed EGDs and poor outcomes in the older age group rather than direct cause and effect.

## Conclusions

Our study demonstrates that patients above the age of 60 are more likely to have a delayed EGD for non-variceal UGIB than patients aged 18-59. Furthermore, the older age group has worse outcomes including in-hospital mortality, blood transfusion requirements, development of acute renal failure, and greater healthcare resource utilization. These findings are independent of hospital and patient characteristics, including patient comorbidities. This study highlights the importance of age considerations when evaluating a patient for a UGIB and the decision to delay intervening with an endoscope. 
